# Supplementary material for: Vertical tearing of subducting plates controlled by geometry and rheology of oceanic plates
Source: Nat Commun. 2023 Dec 1;14:7931. doi: 10.1038/s41467-023-43804-z (PMC10692197; doi:10.1038/s41467-023-43804-z)
Supplement: Supplementary file 1 — Supplementary Information [file 41467_2023_43804_MOESM1_ESM.pdf]

Vertical tearing of subducting plates controlled by geometry and rheology of oceanic plates

Yaguang Chen, Hanlin Chen*, Mingqi Liu*, Taras Gerya*

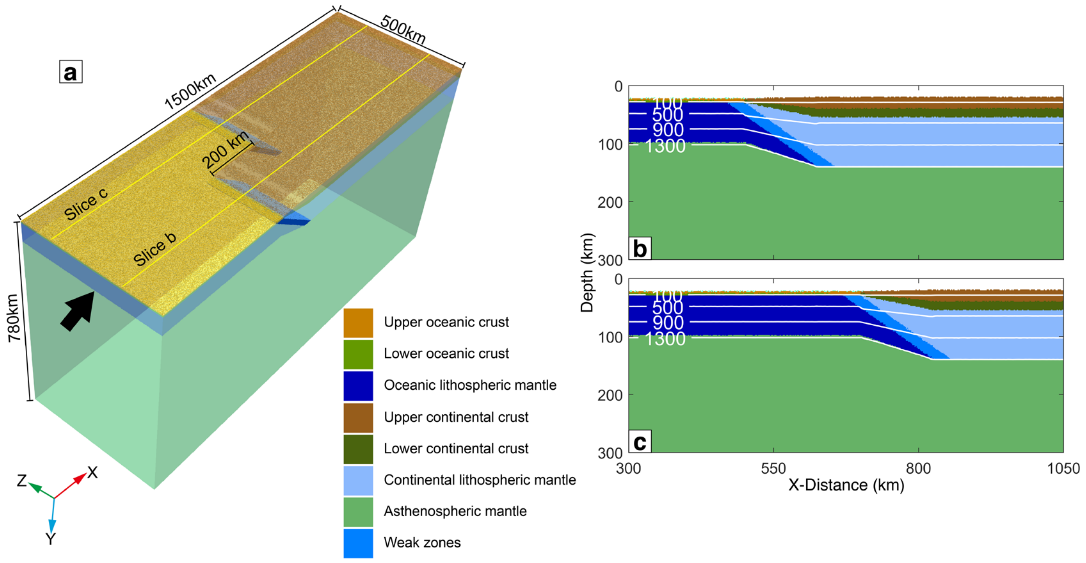

Supplementary Fig. 1 Initial model setup and boundary conditions.

(a) 3D view of the compositional domain of reference model (Mod 12) with a 200 km initial trench offset in the subducting oceanic plate. The bold black arrow on the left ($X = 0$ km) marks the prescribed constant boundary velocity. Yellow lines indicate the two XY slices at (b) $Z = 125$ km and (c) $Z = 375$ km through the two subducting plates, which show the initial temperature and rock material distributions. The white lines represent isotherms in °C, and the oceanic lithosphere thickness is determined by the 1400 K isotherm. Color code for different rock materials is shown at the bottom center of the figure. See Methods for details.

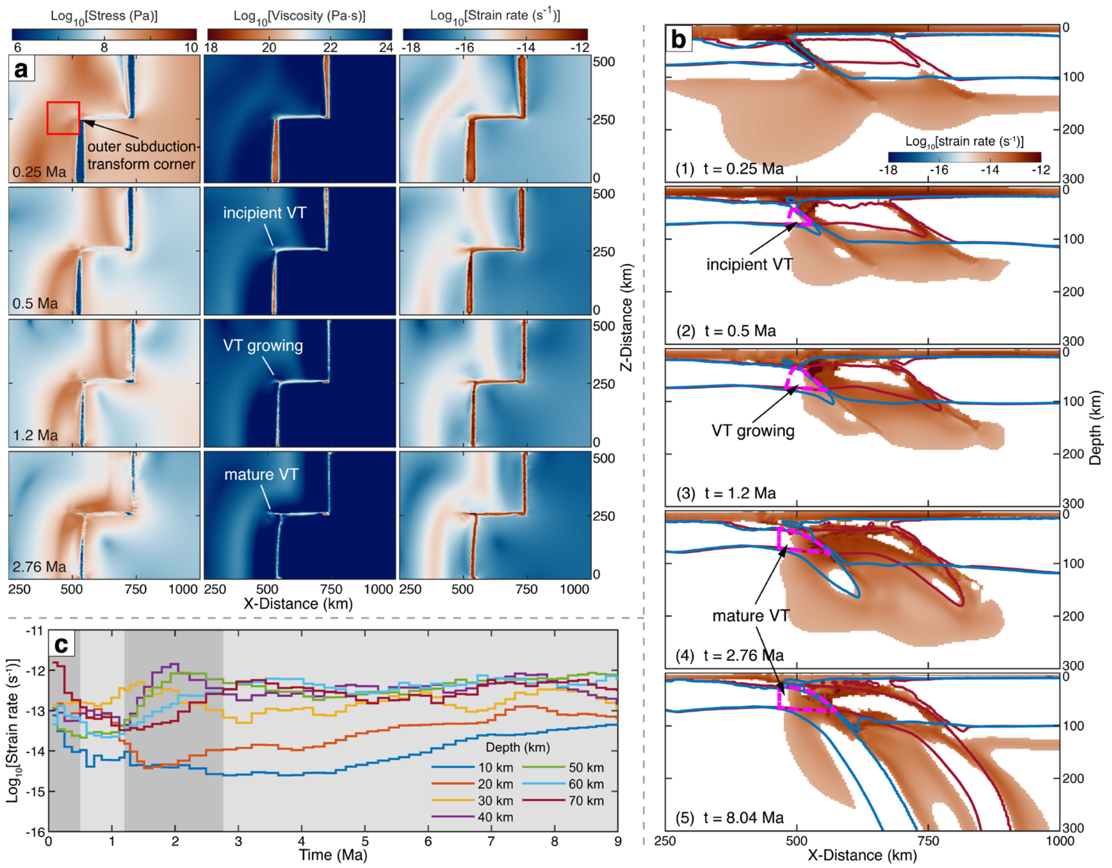

Supplementary Fig. 2 Evolution of vertical tearing in reference model (Mod12).

(a) show horizontal evolution of reference model at different stages (rows): stress concentration stage (from the beginning to 0.5 Ma); incipient VT stage (from 0.5 Ma to 1.2 Ma); VT growing stage (from 1.2 Ma to 2.76 Ma); and mature stage with stably propagating mode-III VT (after 2.76 Ma). The left to right columns shows logarithmic viscosity, second stress invariant, and second strain rate invariant at $Y = 69$ km, respectively. The red hollow square shows the outer subduction-transform corner. (b) Vertical profiles along the VT at different stages. The parts with a high deformation rate at $Z = 250$ km are defined as areas with a strain rate greater than 5×10^{-15} s^{-1}. The blue and red lines are iso-viscosity contours equal to 10^{22} Pa s at $Z = 230$ km and $Z = 270$ km, respectively, representing the shapes of two adjacent subducting plates. Magenta dashed curves refer to the shear regions between these two segments. The high deformation rate parts within these shear regions illustrate the vertical evolution of mode-III VT. Note the stabilization of the VT length after 2.76 Ma. (c) The deformation rate (second invariant of the strain rate) of mode-III VT at different depths, where the mode-III VT is defined as an area with over 80% of the maximum strain rate within the outer subduction-transform corner. The shaded background marks the four evolution stages defined in (a).

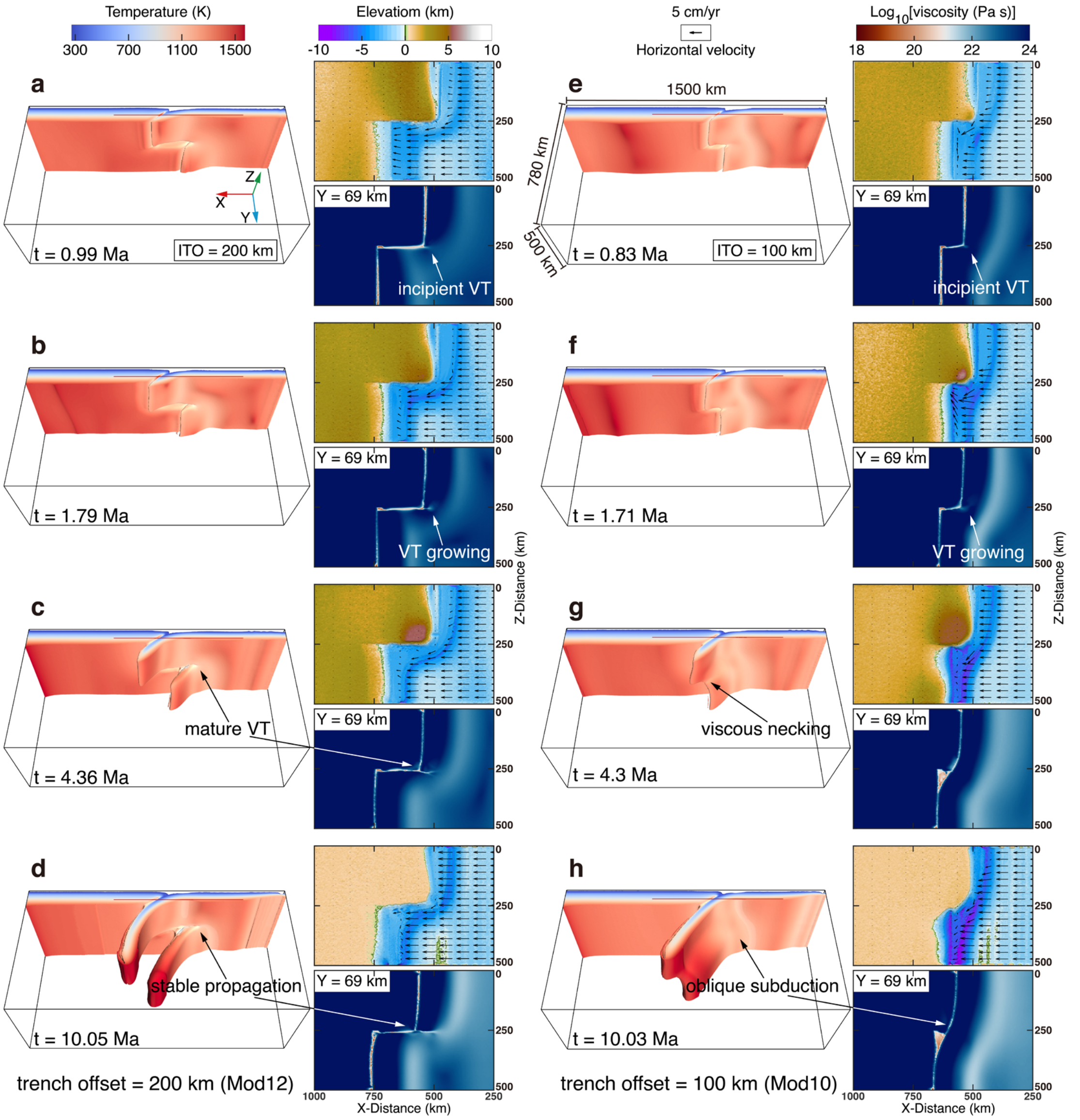

Supplementary Fig. 3 Evolution of models with different initial trench offsets.

(a-d) Results of reference model (Mod12) at different stages as (a) incipient VT, (b) VT growing, (c) mature VT, and then (d) stable propagation, respectively. (e-h) Evolution of Mod10 with a shorter trench offset (100 km). In each section, subducting plate morphology is shown at the left (by the 10^{22} Pa s iso-viscosity contour); the horizontal viscosity slice is at the bottom right, marked by the red line and transparent surface in slab morphology; relative surface elevation is at the top right superimposed by plate velocity at 6-km-depth in arrows representing the crustal deformation. In both models, the ages of subducting oceanic plates are 40 Ma, and strong strain weakening (0.6-0) of the oceanic lithospheric mantle is applied. ITO, initial trench offset.

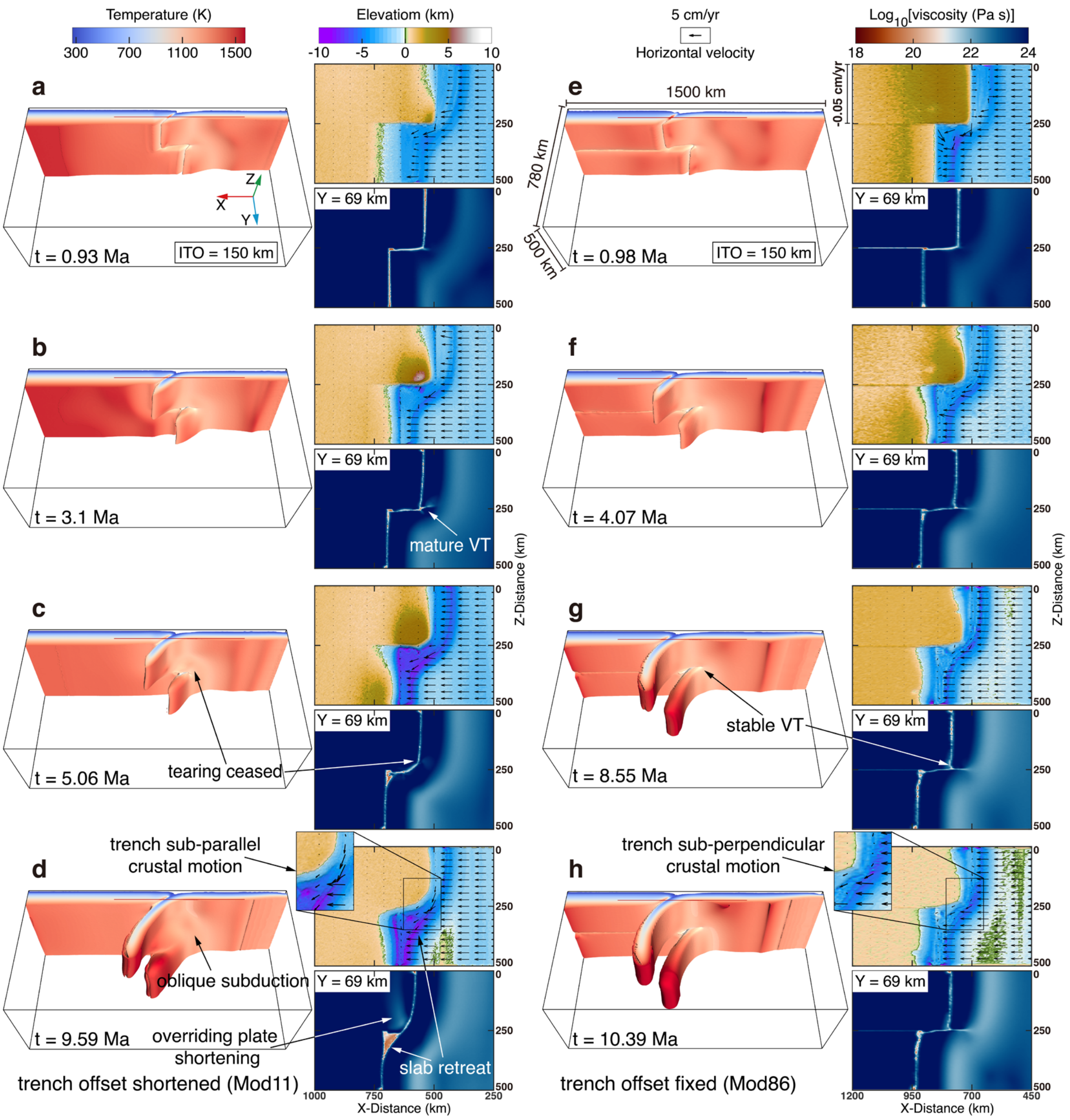

Supplementary Fig. 4 Influence of trench offset shortening on the VT development.

(a-d) Evolution of model with trench offset shortening process. (e-h) Evolution of model without trench offset shortening process. A small convergent velocity (-0.05 cm/yr) is applied on the front half ($Z < 250$ km) of the overriding plate, which can compensate for the shortening process to keep the trench offset stable. In each section, subducting plate morphology is shown at the left (by the 10^{22} Pa s iso-viscosity contour); the horizontal viscosity slice is at the bottom right, marked by the red line and transparent surface in slab morphology; relative surface elevation is at the top right superimposed by plate velocity at 6-km-depth in arrows representing the crustal deformation. In both models, the ages of subducting oceanic plates are 40 Ma, and strong strain weakening (0.6-0) of the oceanic lithospheric mantle is applied. ITO, initial trench offset.

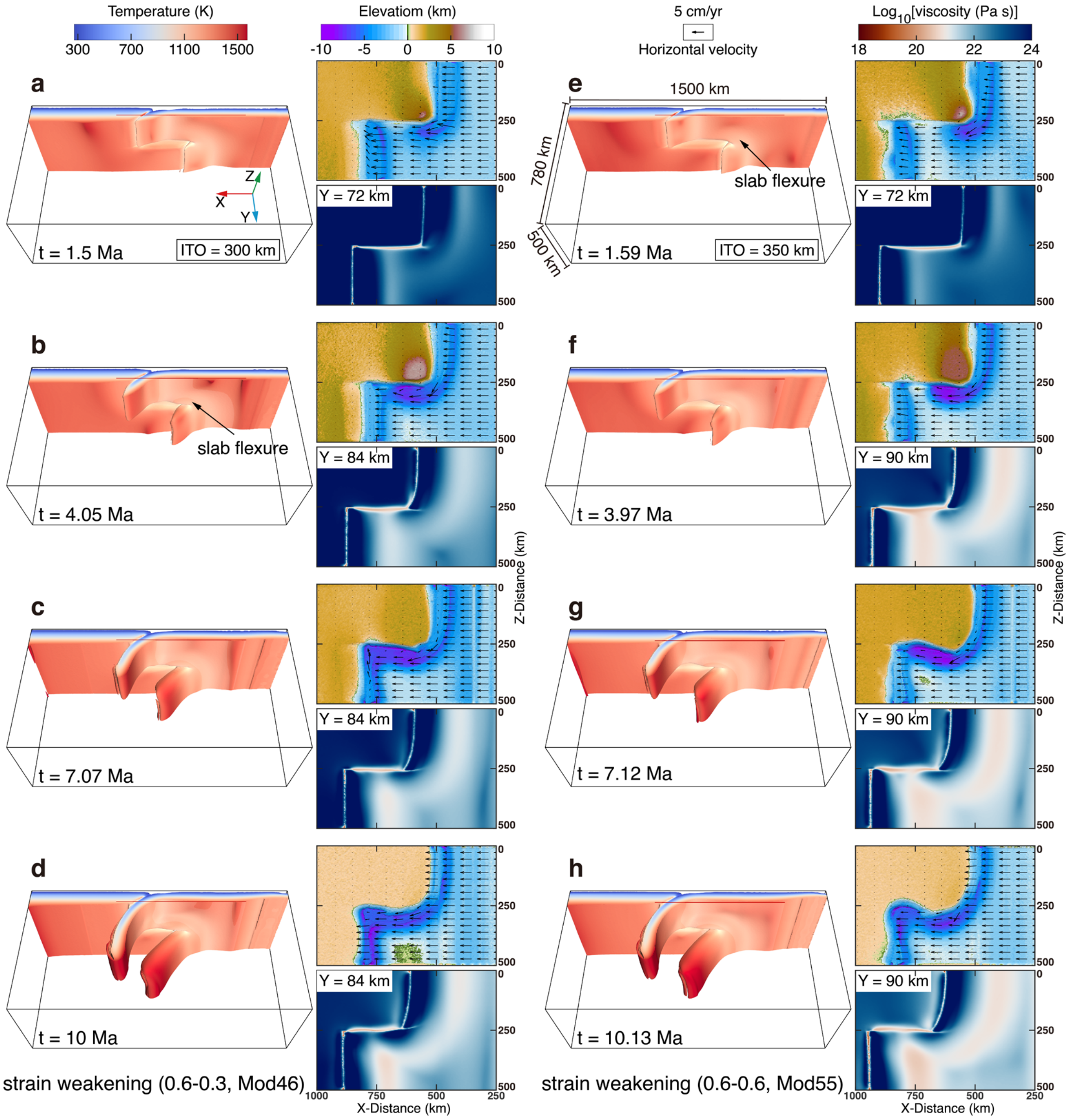

Supplementary Fig. 5 Influence of strain weakening on the VT development.

(a-d) Evolution of model with moderate strain weakening (0.6-0.3). (e-h) Evolution of model without strain weakening (0.6-0.6). In each section, subducting plate morphology is shown at the left (by the 10^{22} Pa s iso-viscosity contour); the horizontal viscosity slice is at the bottom right, marked by the red line and transparent surface in slab morphology; relative surface elevation is at the top right superimposed by plate velocity at 6-km-depth in arrows representing the crustal deformation. Oceanic plate ages are 40 Ma in both models. ITO, initial trench offset.

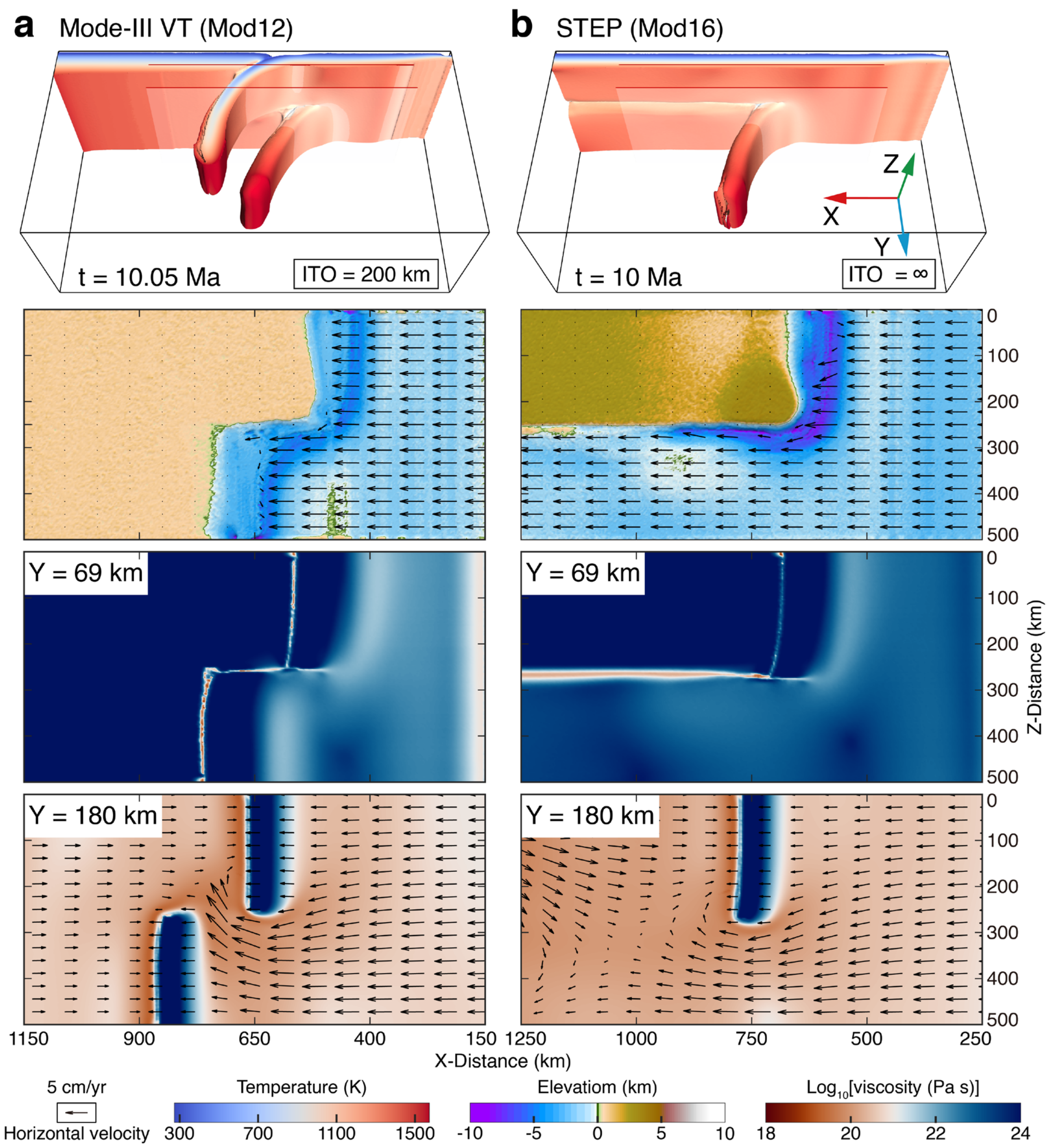

Supplementary Fig. 6 Comparison of model results with mode-III VT and STEP.

Results of the model with (a) two subduction zones and (b) one subduction zone. In each section, subducting plate morphology is shown at the top (by the 10^{22} Pa s iso-viscosity contour); relative surface elevation is at the second row; the third and last rows are the horizontal viscosity slices lithospheric depth (69 km) and asthenospheric depth (180 km). The superimposed velocity arrows on the second and last rows show the crustal deformation at 6 km depth and asthenospheric mantle flow at $Y = 180$ km. ITO, initial trench offset.

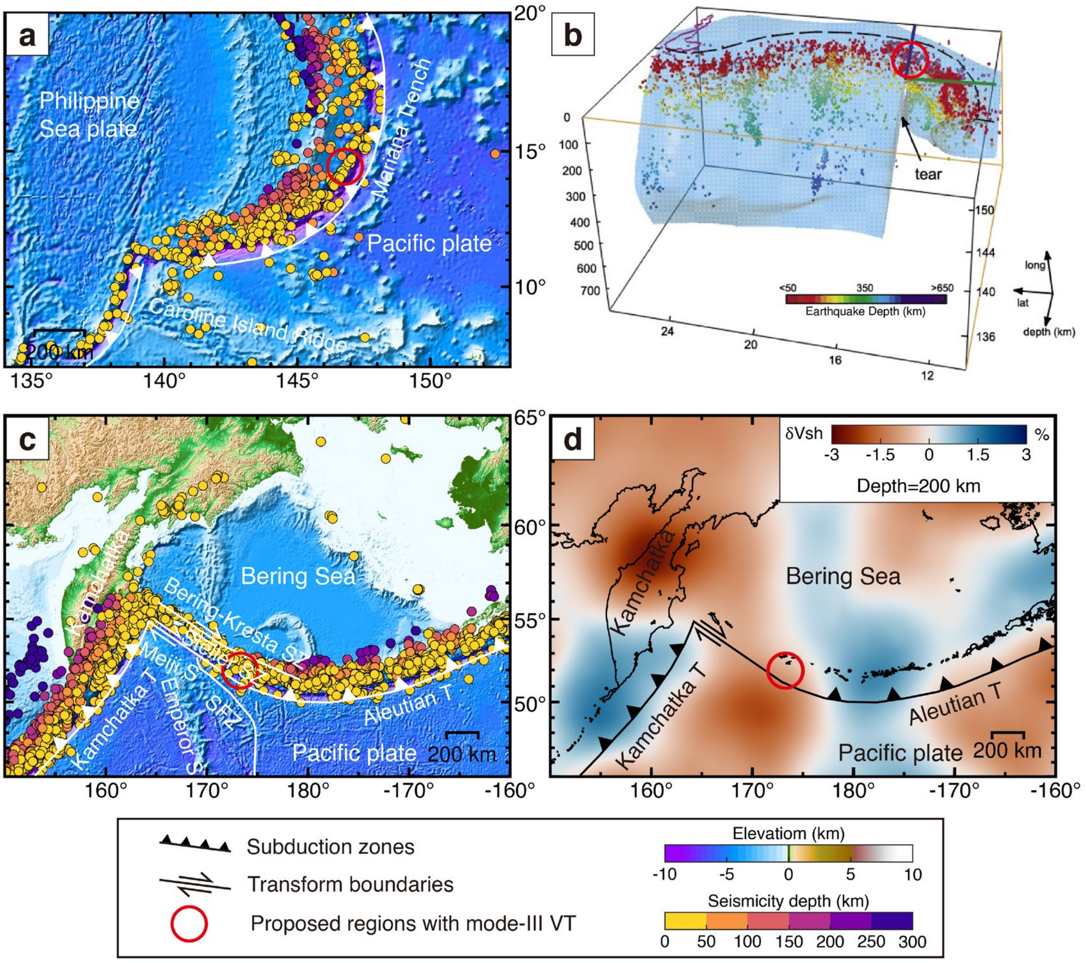

Supplementary Fig. 7 Tectonic maps and geophysical images of regions with mode-III VTs.

Tectonics, topographic maps, seismicity, geophysical images, and locations of mature mode-III VTs of southern Mariana trench[1] (a, b) and Aleutian-Kamchatka trenches[2,3] (c, d). The topography data is from ETOPO1 arc-minute Global Relief Model[4]. Seismicity data (Mw >= 5) in (a, c) is from the Global CMT catalog[5,6]. T, trench; S, seamount; SFZ, Stalemate fracture zone; SZ, shear zone. The slab morphology and the distribution of seismicity in (b) are from Miller et al.[1]. The distribution of seismic velocity anomaly in (d) is based on the tomography model of Thrastarson et al.[7].

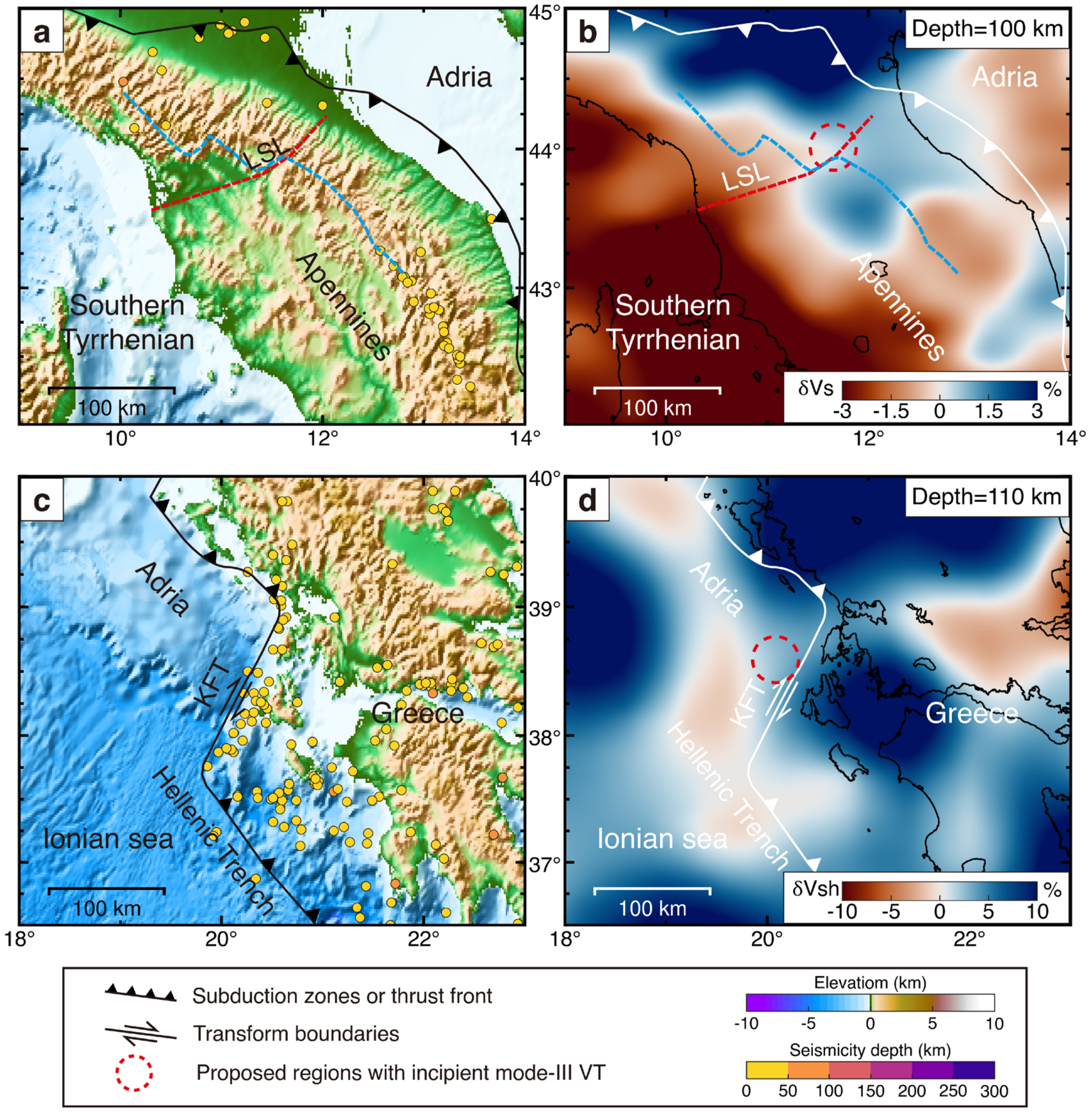

Supplementary Fig. 8 Tectonic maps and seismic tomographic images of regions with incipient mode-III VTs.

Tectonics, topographic maps, seismicity, tomographic images, and locations of incipient mode-III VTs of Apennines regions[8–10] (a, b), and western Hellenic Subduction Zone[11,12] (c, d). The topography data is from ETOPO1 arc-minute Global Relief Model[4]. Seismicity data (Mw >= 5) is from the Global CMT catalog[5,6]. Blue dashed lines in (a, b) refer to the offset drainage divide related to an incipient mode-III VT. LSL, Livorno–Sillaro Lineament; KTF, Kefalonia Transform Fault. The distribution of seismic velocity anomalies in (b, d) are based on the tomography models of El-Sharkawy et al.[10] and Fichtner et al.[12], respectively.

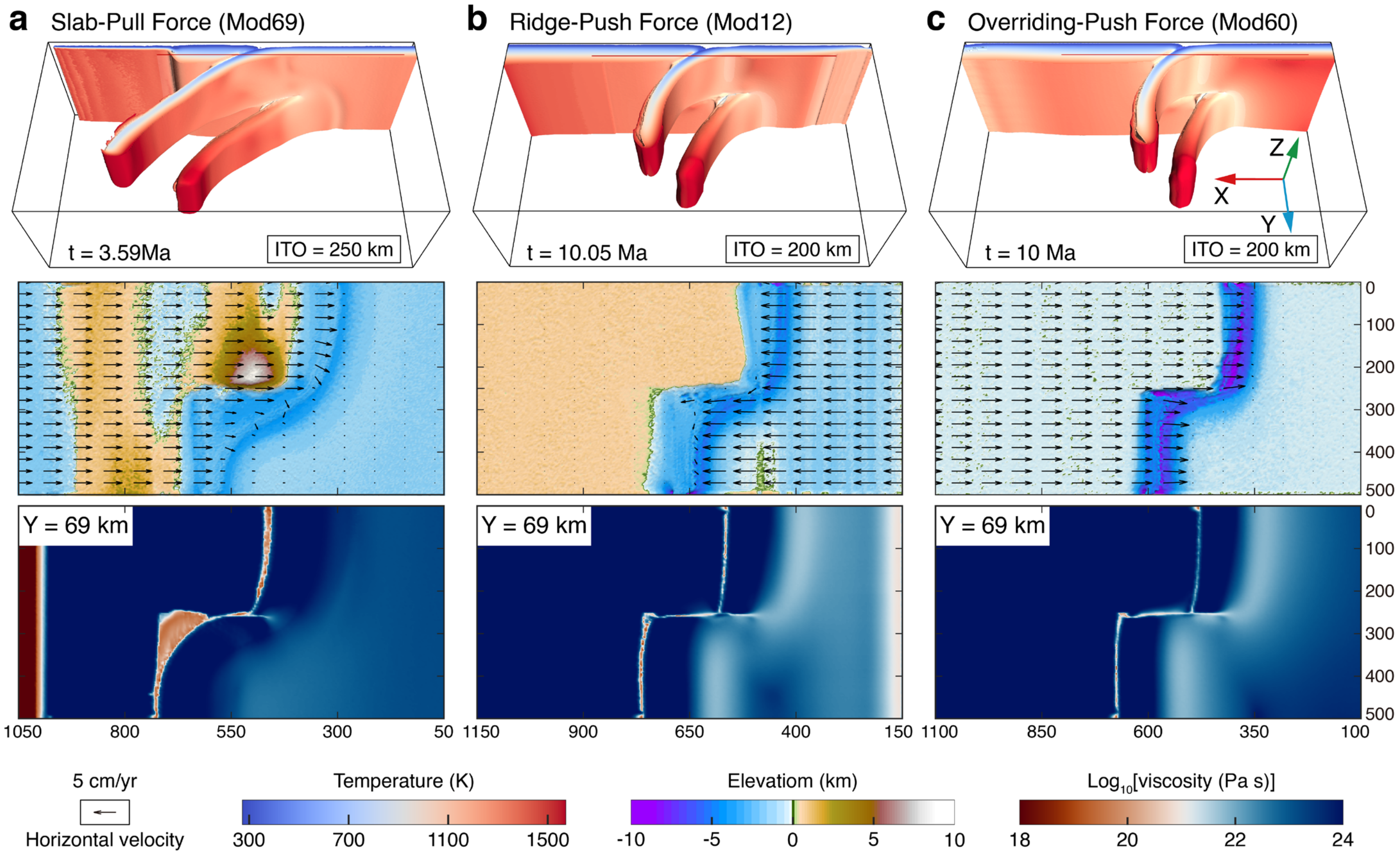

Supplementary Fig. 9 Comparison of model results with variable driven forces.

(a-c) Result of models driven by slab-pull force, ridge-push force, and overriding-plate-push force, respectively. In each section, subducting plate morphology is shown at the top (by the 10^{22} Pa s iso-viscosity contour); the horizontal viscosity slice is at the middle, marked by the red line and transparent surface in slab morphology; relative surface elevation is at the bottom superimposed by plate velocity at 6-km-depth in arrows representing the crustal deformation. In all models, the ages of subducting oceanic plates are 40 Ma, and strong strain weakening (0.6-0) of the oceanic lithospheric mantle is applied. ITO, initial trench offset.

Supplementary Table 1 Summary of approximate locations of proposed generalized mode-III VTs

Region	Abbreviation	Longitude	Latitude	Subdivision*	References
Andaman Arc	AT	91.3	9.8	Mode-III VT	[13]
Carnegie Ridge	CR	-81.57	-1.85	Mode-III VT	[14]
Central-Eastern Hindu-Kush Orogen	CEHKO	71.39	36.25	STEP	[15]
Central-Southern Cocos	CSC	-98.53	15.61	Mode-III VT	[16]
Central-Southern Mariana Trench	CSMT	147.35	14.46	Mode-III VT	[1]
Dabie-Sulu Orogen	DSO	116.5	31	Mode-III VT	[17]
Delhi-Hardwar Ridge	DHR	77.8	30.5	Mode-III VT	[18,19]
Eastern Makran Subduction Zone	EMSZ	65.45	24.57	Mode-III VT	[11,20]
Faizabad Ridge	FR	82.65	27.65	Mode-III VT	[18,19]
Juan Fernandez Ridge	JFR	-73.31	-32.81	Mode-III VT	[21]
Kefalonia Transform Fault	KTF	20.34	38.35	Mode-III VT	[11,22]
Manila Trench	MT	119.29	15.83	Mode-III VT	[23]
Munger-Saharsa Ridge	MSR	87.5	27	Mode-III VT	[18,19]
Nazca Ridge	NR	-77.31	-14.82	Mode-III VT	[24,25]
Northern Apennines	NA	14.01	42.04	Mode-III VT	[8,9]
North-Eastern Calabro-Ionian subduction zone	NECISZ	15.35	37.92	STEP	[26]
North-Eastern Pamir Orogen	NEPO	74.89	39.05	STEP	[27]
North-Eastern Puerto Rico	NEPR	-64.4	19.25	STEP	[28,29]
Northern Gibraltar Arc	NGA	-4	37.15	STEP	[30]
Northern Mariana Trench	NMT	143.04	25.86	Mode-III VT	[31,32]
Northern Sandwich Trench	NST	-30.7	-54.93	STEP	[33]
Northern Sumatra	NS	96.41	1.09	Mode-III VT	[34]
Northern Tonga Trench	NTT	-174.05	-14.5	STEP	[35]
North-Western Puerto Rico	NWPR	-67.39	19.03	STEP	[36,37]
Pliny-Strabo Trenches	PST	28.46	35.8	Mode-III VT	[38,39]
Sandra Ridge	SR	-77.98	5.66	Mode-III VT	[40,41]

Southern Gibraltar Arc	SGA	-4.34	34.91	STEP	42
Southern Lesser Antilles Trench	SLAT	-62.25	10.27	STEP	43
Southern Mariana Trench	SMT	144.1	11.91	Mode-III VT	44
Southern Sandwich Trench	SST	-29.26	-60.73	STEP	45
Southern Solomon Trench	SSolT	162.44	-11.35	Mode-III VT	46
Southern Vanuatu Trench	SVT	171.64	-22.55	STEP	47
South-Western Calabro-Ionian subduction zone	SWCISZ	15.61	38.02	STEP	48,49
South-Western Hikurangi Plateau	SWHP	174.92	-42.27	STEP	50
Western Aleutian Trench	WAT	173	51.66	Mode-III VT	2,3
Western Cyprus Trench	WCT	32.46	35.66	Mode-III VT	38
Western North Sulawesi Trench	WNST	119.52	-0.05	STEP	11,45
Western Ryukyu Trench	WRT	123.3	23.2	Mode-III VT	51

[*]The generalized mode-III VTs subdivided based on their geodynamic settings: STEP develops near the terminations of the subduction zone and separates the subducting and non-subducting plates; Mode-III VT propagates within two subducting segments separating two offset subducting plate sections.

Supplementary Table 2 Conditions and results of the numerical experiments

Model	Trench offset [km]	Plate cooling age; thickness [Ma, km]	φ_0-φ_1 of lithospheric mantle	Boundary velocity [cm/yr]	Result
Mod1	50	20; 53	0.6-0	5	oblique subduction
Mod2	100	20; 53	0.6-0	5	~300 km PL, then oblique subduction
Mod3	150	20; 53	0.6-0	5	stable mode-III VT
Mod4	200	20; 53	0.6-0	5	stable mode-III VT
Mod5	250	20; 53	0.6-0	5	stable mode-III VT
Mod6	300	20; 53	0.6-0	5	stable mode-III VT
Mod7	350	20; 53	0.6-0	5	stable mode-III VT
Mod8	∞ (STEP)	20; 53	0.6-0	5	stable mode-III VT
Mod9	50	40; 75	0.6-0	5	oblique subduction
Mod10	100	40; 75	0.6-0	5	oblique subduction
Mod11	150	40; 75	0.6-0	5	~240 km PL, then oblique subduction
Mod12[a]	200	40; 75	0.6-0	5	stable mode-III VT
Mod13	250	40; 75	0.6-0	5	stable mode-III VT
Mod14	300	40; 75	0.6-0	5	stable mode-III VT
Mod15	350	40; 75	0.6-0	5	stable mode-III VT
Mod16	∞ (STEP)	40; 75	0.6-0	5	stable mode-III VT
Mod17	50	60; 92	0.6-0	5	oblique subduction
Mod18	100	60; 92	0.6-0	5	oblique subduction
Mod19	150	60; 92	0.6-0	5	~160 km PL, then oblique subduction
Mod20	200	60; 92	0.6-0	5	stable mode-III VT
Mod21	250	60; 92	0.6-0	5	stable mode-III VT
Mod22	300	60; 92	0.6-0	5	stable mode-III VT
Mod23	350	60; 92	0.6-0	5	stable mode-III VT
Mod24	∞ (STEP)	60; 92	0.6-0	5	stable mode-III VT
Mod25	50	80; 107	0.6-0	5	oblique subduction
Mod26	100	80; 107	0.6-0	5	oblique subduction
Mod27	150	80; 107	0.6-0	5	oblique subduction
Mod28	200	80; 107	0.6-0	5	~230 km PL, then oblique subduction

Mod29	250	80; 107	0.6-0	5	stable mode-III VT
Mod30	300	80; 107	0.6-0	5	stable mode-III VT
Mod31	350	80; 107	0.6-0	5	stable mode-III VT
Mod32	∞ (STEP)	80; 107	0.6-0	5	stable mode-III VT
Mod33	50	100; 119	0.6-0	5	oblique subduction
Mod34	100	100; 119	0.6-0	5	oblique subduction
Mod35	150	100; 119	0.6-0	5	oblique subduction
Mod36	200	100; 119	0.6-0	5	~70 km PL, then oblique subduction
Mod37	250	100; 119	0.6-0	5	~200 km PL, then oblique subduction
Mod38	300	100; 119	0.6-0	5	~290 km PL, then oblique subduction
Mod39	350	100; 119	0.6-0	5	stable mode-III VT
Mod40	∞ (STEP)	100; 119	0.6-0	5	stable mode-III VT
Mod41	50	40; 75	0.6-0.3	5	oblique subduction
Mod42	100	40; 75	0.6-0.3	5	oblique subduction
Mod43	150	40; 75	0.6-0.3	5	~70 km PL, then oblique subduction
Mod44	200	40; 75	0.6-0.3	5	~140 km PL, then oblique subduction
Mod45	250	40; 75	0.6-0.3	5	~410 km PL, then oblique subduction
Mod46	300	40; 75	0.6-0.3	5	stable mode-III VT
Mod47	350	40; 75	0.6-0.3	5	stable mode-III VT
Mod48	∞ (STEP)	40; 75	0.6-0.3	5	stable mode-III VT
Mod49	50	40; 75	0.6-0.6	5	oblique subduction
Mod50	100	40; 75	0.6-0.6	5	oblique subduction
Mod51	150	40; 75	0.6-0.6	5	oblique subduction
Mod52	200	40; 75	0.6-0.6	5	~110 km PL, then oblique subduction
Mod53	250	40; 75	0.6-0.6	5	~240 km PL, then oblique subduction
Mod54	300	40; 75	0.6-0.6	5	~340 km PL, then oblique subduction
Mod55	350	40; 75	0.6-0.6	5	stable mode-III VT
Mod56	∞ (STEP)	40; 75	0.6-0.6	5	stable mode-III VT

Mod57[b]	50	40; 75	0.6-0	-5	oblique subduction
Mod58[b]	100	40; 75	0.6-0	-5	~100 km PL, then oblique subduction
Mod59[b]	150	40; 75	0.6-0	-5	~360 km PL, then oblique subduction
Mod60[b]	200	40; 75	0.6-0	-5	stable mode-III VT
Mod61[b]	250	40; 75	0.6-0	-5	stable mode-III VT
Mod62[b]	300	40; 75	0.6-0	-5	stable mode-III VT
Mod63[b]	350	40; 75	0.6-0	-5	stable mode-III VT
Mod64[b]	∞ (STEP)	40; 75	0.6-0	-5	stable mode-III VT
Mod65[c]	50	40; 75	0.6-0	0	oblique subduction
Mod66[c]	100	40; 75	0.6-0	0	oblique subduction
Mod67[c]	150	40; 75	0.6-0	0	~110 km PL, then oblique subduction
Mod68[c]	200	40; 75	0.6-0	0	~320 km PL, then oblique subduction
Mod69[c]	250	40; 75	0.6-0	0	stable mode-III VT
Mod70[c]	300	40; 75	0.6-0	0	stable mode-III VT
Mod71[c]	350	40; 75	0.6-0	0	stable mode-III VT
Mod72[c]	∞ (STEP)	40; 75	0.6-0	0	stable mode-III VT
Mod73[c]	150	20; 53	0.6-0	0	slab break-off
Mod74	50	40; 75	0.6-0	2	oblique subduction
Mod75	100	40; 75	0.6-0	2	oblique subduction
Mod76	150	40; 75	0.6-0	2	~150 km PL, then oblique subduction
Mod77	200	40; 75	0.6-0	2	~280 km PL, then oblique subduction
Mod78	250	40; 75	0.6-0	2	stable mode-III VT
Mod79	300	40; 75	0.6-0	2	stable mode-III VT
Mod80	50	40; 75	0.6-0	8	oblique subduction
Mod81	100	40; 75	0.6-0	8	~200 km PL, then oblique subduction
Mod82	150	40; 75	0.6-0	8	~370 km PL, then oblique subduction
Mod83	200	40; 75	0.6-0	8	stable mode-III VT
Mod84	250	40; 75	0.6-0	8	stable mode-III VT
Mod85	300	40; 75	0.6-0	8	stable mode-III VT

Mod86[d]	150	40; 75	0.6-0	5	stable mode-III VT, and trench offset remain constant
Mod87[e]	150	40; 75	0.6-0	5	stable mode-III VT, and trench offset lengthening
Mod88[f]	100	40; 75	0.6-0	5	oblique subduction
Mod89[f]	150	40; 75	0.6-0	5	~320 km PL, then oblique subduction
Mod90[f]	200	40; 75	0.6-0	5	stable mode-III VT
Mod91[g]	100	40; 75	0.6-0	5	oblique subduction
Mod92[g]	150	40; 75	0.6-0	5	~170 km PL, then oblique subduction
Mod93[g]	200	40; 75	0.6-0	5	stable mode-III VT
Mod94[f]	300	40; 75	0.6-0.6	5	oblique subduction
Mod95[f]	350	40; 75	0.6-0.6	5	~200 km PL, then oblique subduction
Mod96[f]	400	40; 75	0.6-0.6	5	stable mode-III VT
Mod97[g]	300	40; 75	0.6-0.6	5	stable mode-III VT
Mod98[g]	350	40; 75	0.6-0.6	5	stable mode-III VT
Mod99[g]	400	40; 75	0.6-0.6	5	stable mode-III VT

Model results are divided into three types according to the propagation length (PL) of mode-III VT: oblique subduction mode, where the PL is less than 130 km; transition mode, where the PL is between 130 km and 500 km; and tearing mode, where the mode-III VT can propagate over 500 km.

[a]Reference model.

[b]Models driven by the overriding plate-push force.

[c]Models driven by the slab-pull force.

[d]Model with -0.05 cm/yr convergent velocity on the front half ($Z < 250$ km) of the overriding plate, compensating the trench offset shortening rate to keep the trench offset at a constant.

[e]Model with -0.25 cm/yr convergent velocity on the front half ($Z < 250$ km) of the overriding plate to generate a trench offset lengthening.

[f]Models with 7.08 km resolution (lower than the reference model) along the x-direction.

[g]Models with 2.60 km resolution (higher than the reference model) along the x-direction.

Supplementary Table 3 Physical properties of rock materials used in numerical models

Material	ρ_0 [kg/m^3]	k [W/m/K, at T_k]	A_D [Pa^{n}s]	n	E [kJ/mol]	V [J/mol/MPa]	σ_{cr} [Pa]	C_0 [Mpa]	φ_0	φ_1	Rheology/ flow law	H_r [μW/m^3]
Upper continental crust	2750	0.64+807/(T+77)	1.97×10^{17}	2.3	154	0	3×10^4	3	0.6	0.6	wet quartzite	2
Lower continental crust	2950	1.18+474/(T+77)	4.80×10^{22}	3.2	238	0	3×10^4	3	0.6	0.6	plagioclase An_{75}	0.2
Oceanic upper crust	3000	1.18+474/(T+77)	4.80×10^{22}	3.2	238	0	3×10^4	3	0	0	plagioclase An_{75}	0.22
Oceanic lower crust	3000	1.18+474/(T+77)	4.80×10^{22}	3.2	238	0	3×10^4	3	0.6	0.3	plagioclase An_{75}	0.24
Lithospheric mantle	3300	0.73+1293/(T+77)	3.98×10^{16}	3.5	532	12	3×10^4	3	0.6	Varied* (0.6-0)	dry olivine	0.022
Asthenosphere	3300	0.73+1293/(T+77)	3.98×10^{16}	3.5	532	12	3×10^4	3	0.6	0	dry olivine	0.022
Weak zone	3300	0.73+1293/(T+77)	5.01×10^{20}	4	470	8	3×10^7	3	0	0	wet olivine	0.022
Reference	[52]	[53]	[54]	[54]	[54]	-	-	-	-	-	[54]	[55]

ρ_0 is the initial density, k is the thermal conductivity, A_D is the pre-exponential factor, n is the stress exponent of the viscous creep, E is the activation energy, V is the activation volume, σ_{cr} is the diffusion–dislocation creeps transition stress, C_0 is the material cohesion, φ_0 and φ_1 are the initial and final internal friction coefficient, respectively, and H_r is the radiogenic heat production. *The final internal friction coefficient of the oceanic lithospheric mantle is varied in different models (Supplementary Table 2) to control the magnitude of brittle/plastic strain weakening, as strong (0), moderate (0.3), and no (0.6) strain weakening. For the continental lithospheric mantle, the final internal friction coefficient is 0.3 in all models.

Supplementary References

1. Miller, M. S., Gorbatov, A. & Kennett, B. L. N. Three-dimensional visualization of a near-vertical slab tear beneath the southern Mariana arc. *Geochem. Geophys. Geosystems* **7**, (2006).
2. Yogodzinski, G. M. *et al.* Geochemical evidence for the melting of subducting oceanic lithosphere at plate edges. *Nature* **409**, 500–504 (2001).
3. Levin, V., Shapiro, N. M., Park, J. & Ritzwoller, M. H. Slab portal beneath the western Aleutians. *Geology* **33**, 253–256 (2005).
4. Amante, C. & Eakins, B. ETOPO1 1 Arc-Minute Global Relief Model: procedures, data sources and analysis. in *NOAA Technical Memorandum NESDIS NGDC-24* (National Geophysical Data Center, 2009). doi:10.7289/V5C8276M.
5. Dziewonski, A. M., Chou, T.-A. & Woodhouse, J. H. Determination of earthquake source parameters from waveform data for studies of global and regional seismicity. *J. Geophys. Res. Solid Earth* **86**, 2825–2852 (1981).
6. Ekström, G., Nettles, M. & Dziewoński, A. M. The global CMT project 2004–2010: Centroid-moment tensors for 13,017 earthquakes. *Phys. Earth Planet. Inter.* **200–201**, 1–9 (2012).
7. Thrastarson, S. *et al.* Data-adaptive global full-waveform inversion. *Geophys. J. Int.* **230**, 1374–1393 (2022).
8. Piana Agostinetti, N. The structure of the Moho in the Northern Apennines: Evidence for an incipient slab tear fault? *Tectonophysics* **655**, 88–96 (2015).
9. Rosenbaum, G. & Agostinetti, N. P. Crustal and upper mantle responses to lithospheric segmentation in the northern Apennines. *Tectonics* **34**, 648–661 (2015).
10. El-Sharkawy, A. *et al.* The Slab Puzzle of the Alpine-Mediterranean Region: Insights From a New, High-Resolution, Shear Wave Velocity Model of the Upper Mantle. *Geochem. Geophys. Geosystems* **21**, e2020GC008993 (2020).
11. Özbakır, A. D., Govers, R. & Fichtner, A. The Kefalonia Transform Fault: A STEP fault in the making. *Tectonophysics* **787**, 228471 (2020).

12. Fichtner, A. *et al.* The Collaborative Seismic Earth Model: Generation 1. *Geophys. Res. Lett.* **45**, 4007–4016 (2018).

13. Kumar, P., Srijayanthi, G. & Kumar, M. R. Seismic evidence for tearing in the subducting Indian slab beneath the Andaman arc. *Geophys. Res. Lett.* **43**, 4899–4904 (2016).

14. Gutscher, M.-A., Malavieille, J., Lallemand, S. & Collot, J.-Y. Tectonic segmentation of the North Andean margin: impact of the Carnegie Ridge collision. *Earth Planet. Sci. Lett.* **168**, 255–270 (1999).

15. Sippl, C. *et al.* Geometry of the Pamir-Hindu Kush intermediate-depth earthquake zone from local seismic data: EARTHQUAKE DISTRIBUTION PAMIR-HINDU KUSH. *J. Geophys. Res. Solid Earth* **118**, 1438–1457 (2013).

16. Dougherty, S. L. & Clayton, R. W. Seismicity and structure in central Mexico: Evidence for a possible slab tear in the South Cocos plate. *J. Geophys. Res. Solid Earth* **119**, 3424–3447 (2014).

17. Zhao, T., Zhu, G., Lin, S. & Wang, H. Indentation-induced tearing of a subducting continent: Evidence from the Tan–Lu Fault Zone, East China. *Earth-Sci. Rev.* **152**, 14–36 (2016).

18. Godin, L. & Harris, L. B. Tracking basement cross-strike discontinuities in the Indian crust beneath the Himalayan orogen using gravity data – relationship to upper crustal faults. *Geophys. J. Int.* **198**, 198–215 (2014).

19. Sunilkumar, T. C., Earnest, A., Silpa, K. & Andrews, R. Rupture of the Indian Slab in the 2011 Mw 6.9 Sikkim Himalaya Earthquake and Its Tectonic Implications. *J. Geophys. Res. Solid Earth* **124**, 2623–2637 (2019).

20. Husson, L. *et al.* Dynamic ups and downs of the Himalaya. *Geology* **42**, 839–842 (2014).

21. Pesicek, J. D., Engdahl, E. R., Thurber, C. H., DeShon, H. R. & Lange, D. Mantle subducting slab structure in the region of the 2010 M8.8 Maule earthquake (30–40°S), Chile. *Geophys. J. Int.* **191**, 317–324 (2012).

22. Suckale, J. *et al.* High-resolution seismic imaging of the western Hellenic subduction zone using teleseismic scattered waves. *Geophys. J. Int.* **178**, 775–791 (2009).

23. Fan, J., Wu, S. & Spence, G. Tomographic evidence for a slab tear induced by fossil ridge subduction at Manila Trench, South China Sea. *Int. Geol. Rev.* **57**, 998–1013 (2015).

24. Scire, A. *et al.* Imaging the transition from flat to normal subduction: variations in the structure of the Nazca slab and upper mantle under southern Peru and northwestern Bolivia. *Geophys. J. Int.* **204**, 457–479 (2016).

25. Antonijevic, S. K. *et al.* Effects of change in slab geometry on the mantle flow and slab fabric in Southern Peru. *J. Geophys. Res. Solid Earth* **121**, 7252–7270 (2016).

26. Miller, M. S. & Agostinetti, N. P. Erosion of the continental lithosphere at the cusps of the Calabrian arc: Evidence from S receiver functions analysis. *Geophys. Res. Lett.* **38**, L23301 (2011).

27. Sobel, E. R. *et al.* Oceanic-style subduction controls late Cenozoic deformation of the Northern Pamir orogen. *Earth Planet. Sci. Lett.* **363**, 204–218 (2013).

28. Brink, U. ten. Vertical motions of the Puerto Rico Trench and Puerto Rico and their cause. *J. Geophys. Res. Solid Earth* **110**, B06404 (2005).

29. Meighan, H. E., Pulliam, J., Brink, U. ten & López-Venegas, A. M. Seismic evidence for a slab tear at the Puerto Rico Trench. *J. Geophys. Res. Solid Earth* **118**, 2915–2923 (2013).

30. Mancilla, F. de L. *et al.* Slab rupture and delamination under the Betics and Rif constrained from receiver functions. *Tectonophysics* **663**, 225–237 (2015).

31. Zhao, D., Fujisawa, M. & Toyokuni, G. Tomography of the subducting Pacific slab and the 2015 Bonin deepest earthquake (Mw 7.9). *Sci. Rep.* **7**, 44487 (2017).

32. Kong, X. *et al.* Causes of earthquake spatial distribution beneath the Izu-Bonin-Mariana Arc. *J. Asian Earth Sci.* **151**, 90–100 (2018).

33. Leat, P. T. *et al.* Magma genesis and mantle flow at a subducting slab edge: the

South Sandwich arc-basin system. *Earth Planet. Sci. Lett.* **227**, 17–35 (2004).

34. Liu, S. *et al.* Slab Morphology Beneath Northern Sumatra Revealed by Regional and Teleseismic Traveltime Tomography. *J. Geophys. Res. Solid Earth* **124**, 10544–10564 (2019).

35. Millen, D. W. & Hamburger, M. W. Seismological evidence for tearing of the Pacific plate at the northern termination of the Tonga subduction zone. *Geology* **26**, 659–662 (1998).

36. Harris, C. W., Miller, M. S. & Porritt, R. W. Tomographic Imaging of Slab Segmentation and Deformation in the Greater Antilles. *Geochem. Geophys. Geosystems* **19**, 2292–2307 (2018).

37. Braszus, B. *et al.* Subduction history of the Caribbean from upper-mantle seismic imaging and plate reconstruction. *Nat. Commun.* **12**, 4211 (2021).

38. Biryol, C. B., Beck, S. L., Zandt, G. & Özacar, A. A. Segmented African lithosphere beneath the Anatolian region inferred from teleseismic P-wave tomography. *Geophys. J. Int.* **184**, 1037–1057 (2011).

39. Salaün, G. *et al.* High-resolution surface wave tomography beneath the Aegean-Anatolia region: constraints on upper-mantle structure. *Geophys. J. Int.* **190**, 406–420 (2012).

40. Vargas, C. A. & Mann, P. Tearing and Breaking Off of Subducted Slabs as the Result of Collision of the Panama Arc-Indenter with Northwestern South America. *Bull. Seismol. Soc. Am.* **103**, 2025–2046 (2013).

41. Wagner, L. S. *et al.* Transient slab flattening beneath Colombia. *Geophys. Res. Lett.* **44**, 6616–6623 (2017).

42. Spakman, W., Chertova, M. V., van den Berg, A. & van Hinsbergen, D. J. J. Puzzling features of western Mediterranean tectonics explained by slab dragging. *Nat. Geosci.* **11**, 211–216 (2018).

43. Clark, S. A. *et al.* Identification and tectonic implications of a tear in the South American plate at the southern end of the Lesser Antilles. *Geochem. Geophys.*

Geosystems **9**, (2008).

44. Gvirtzman, Z. & Stern, R. J. Bathymetry of Mariana trench-arc system and formation of the Challenger Deep as a consequence of weak plate coupling. *Tectonics* **23**, (2004).

45. Govers, R. & Wortel, M. J. R. Lithosphere tearing at STEP faults: response to edges of subduction zones. *Earth Planet. Sci. Lett.* **236**, 505–523 (2005).

46. Neely, J. S. & Furlong, K. P. Evidence of displacement-driven maturation along the San Cristobal Trough transform plate boundary. *Earth Planet. Sci. Lett.* **485**, 88–98 (2018).

47. Martin, A. K. Concave slab out board of the Tonga subduction zone caused by opposite toroidal flows under the North Fiji Basin. *Tectonophysics* **622**, 56–61 (2014).

48. Gutscher, M.-A. *et al.* Tectonic expression of an active slab tear from high-resolution seismic and bathymetric data offshore Sicily (Ionian Sea). *Tectonics* **35**, 39–54 (2016).

49. Scarfì, L. *et al.* Slab narrowing in the Central Mediterranean: the Calabro-Ionian subduction zone as imaged by high resolution seismic tomography. *Sci. Rep.* **8**, 5178 (2018).

50. Reyners, M. The central role of the Hikurangi Plateau in the Cenozoic tectonics of New Zealand and the Southwest Pacific. *Earth Planet. Sci. Lett.* **361**, 460–468 (2013).

51. Lin, J.-Y., Hsu, S.-K. & Sibuet, J.-C. Melting features along the western Ryukyu slab edge (northeast Taiwan): Tomographic evidence. *J. Geophys. Res. Solid Earth* **109**, (2004).

52. Munch, J., Gerya, T. & Ueda, K. Oceanic crust recycling controlled by weakening at slab edges. *Nat. Commun.* **11**, 2009 (2020).

53. Gerya, T. V., Stern, R. J., Baes, M., Sobolev, S. V. & Whattam, S. A. Plate tectonics on the Earth triggered by plume-induced subduction initiation. *Nature* **527**, 221–225 (2015).

54. Ranalli, G. *Rheology of the Earth*. (Chapman & Hall, 1995).

55. Balázs, A. *et al.* Oblique subduction and mantle flow control on upper plate deformation: 3D geodynamic modeling. *Earth Planet. Sci. Lett.* **569**, 117056 (2021).
